# Workplace ostracism in nursing: a scoping review

**DOI:** 10.3389/fpubh.2026.1884011

**Published:** 2026-07-16

**Authors:** Shiya Hu, Binfen Lin, Fenglan Lin

**Affiliations:** 1 Department of Education, The Affiliated People's Hospital of Fujian University of Traditional Chinese Medicine, Fuzhou, China; 2 School of Nursing, Fujian University of Traditional Chinese Medicine, Fuzhou, China; 3 Department of Respiratory and Critical Care Medicine, Lishui Central Hospital, Lishui, China

**Keywords:** nurses, nursing management, scoping review, work environment, workplace ostracism

## Abstract

**Background:**

Workplace ostracism is a negative workplace experience. It can affect nurses' psychological wellbeing and work-related behaviors. However, this issue has not been fully discussed in nursing, a profession characterized by intensive interpersonal interaction and complex social contexts.

**Objective:**

This scoping review aimed to examine studies on workplace ostracism in nursing. It summarized the current research status, variable roles, potential mechanisms, and major outcomes.

**Methods:**

This study followed the scoping review guidance of the Joanna Briggs Institute. PubMed, Embase, Web of Science, APA PsycInfo, The Cochrane Library, Scopus, CINAHL, and MEDLINE Ultimate were searched. The search covered the period from database inception to April 19, 2026. Two Reviewers independently screened the studies, reviewed the full texts, and extracted the data.

**Results:**

A total of 21 studies from nine countries were included. Most studies were conducted in China, Egypt, and Pakistan, indicating a degree of geographical concentration in the current evidence base. The included studies were predominantly cross-sectional quantitative surveys, with only a small number using time-lagged designs, multilevel nested analyses, or qualitative interviews, suggesting limited methodological diversity. In the field of nursing, workplace exclusion is mainly studied as an independent variable, mediating variable, or outcome variable. The correlates of workplace ostracism among nurses involved individual and organizational factors. Potential mechanisms included psychological resource depletion, reduced organizational identification, and impaired communication. Associated outcomes covered multiple levels, including individual psychological health, professional behavior, and organizational functioning.

**Conclusion:**

Current evidence suggests that workplace ostracism is a complex and context-dependent phenomenon in nursing organizations. Given the predominance of cross-sectional designs, future research should strengthen theoretical integration and methodological development, including longitudinal studies, intervention studies, mixed-methods research, and cross-cultural comparisons, to further clarify its pathways and effective intervention strategies. Nursing managers should address leadership behaviors, team culture, communication safety, and organizational support to reduce workplace ostracism and its potential adverse consequences.

## 1 Introduction

With population aging and the increasing burden of chronic diseases, the global demand for nursing services continues to rise (1). A healthy work environment is important for nurses' job satisfaction. It also affects care quality, patient safety, and nurses' intention to remain in the profession (2). However, nursing work is often characterized by high workload, high emotional demands, and strong team dependence. In clinical practice, nurses are required to perform complex care tasks. They are also expected to provide emotional support to patients and their families to reduce negative emotions (3). Under these conditions, nurses may be exposed to toxic workplace environments (4). These environments may include workplace ostracism, workplace bullying, and workplace violence. Such negative workplace experiences may further increase nurses' psychological burden, weaken their work engagement, and ultimately affect the quality of nursing care.

Workplace ostracism has received increasing attention in the nursing field. It is commonly defined as an employee's perception of being ignored or excluded by others in the workplace, including colleagues, supervisors, or subordinates (5). Compared with overt forms of mistreatment, such as workplace violence and bullying, workplace ostracism is often more subtle. It may appear as silent treatment, information exclusion, relational distancing, or exclusion from team interactions (6). Therefore, it is often difficult to identify and address. Although workplace ostracism is usually hidden, nurses may still perceive a relatively high level of ostracism in clinical settings (7).

Healthcare institutions are important social environments where nurses interact with other healthcare professionals and patients. When nurses feel ignored, excluded, or marginalized within a team, their psychological safety may be reduced. Their sense of organizational belonging and work engagement may also be weakened. Previous studies have shown that workplace ostracism can affect nurses' behaviors and mental health (8). It has been associated with stress (3), emotional dysregulation (9), and loneliness (10). These factors may further have negative effects on work quality (9), work-related behaviors (11), and intention to stay (12). Therefore, workplace ostracism among nurses is not only an individual interpersonal experience. It may also become an organizational risk factor that affects team functioning and the quality of nursing services.

From a theoretical perspective, workplace ostracism can be understood as a workplace social stressor characterized by resource depletion and relational disruption. Conservation of Resources Theory (13) suggests that when individuals' resources are threatened or lost, they strive to protect, maintain, and rebuild their existing resources in order to cope with potential future resource loss. When nurses experience workplace ostracism, their emotion regulation capacity may be depleted, and they may engage in maladaptive behaviors to protect their remaining interpersonal resources (14). Social Exchange Theory (15) emphasizes that individuals tend to respond to others in a manner that is similar or equivalent to how they are treated. Therefore, when employees experience ostracism at work, their reciprocal trust in colleagues, teams, and organizations may be undermined, which may further affect their work attitudes and behavioral responses (16). In addition, Belongingness Theory (17) proposes that individuals have a fundamental psychological need to maintain stable social connections and gain group acceptance. A toxic environment characterized by workplace ostracism may threaten nurses' sense of organizational belonging and psychological safety.

Against this theoretical background, workplace ostracism among nurses is not merely a negative interpersonal experience, but also an important organizational phenomenon that may influence nurses' psychological health, work attitudes, and nursing behaviors through resource depletion, impaired belongingness, and disrupted social exchange relationships. In recent years, studies on workplace ostracism among nurses have gradually increased. Some studies have examined workplace ostracism as an independent variable. These studies explored its effects on nurses' mental health, work attitudes, and behaviors (3, 9, 18). Other studies have treated workplace ostracism as a mediating variable. These studies explained the relationships between leadership behaviors, team factors, individual traits, and work outcomes (19, 20). In addition, some studies have examined workplace ostracism as an outcome variable. These studies analyzed how organizational environments and interpersonal factors may contribute to workplace ostracism (21, 22). However, there is still a lack of systematic synthesis of the research status, variable relationships, and evidence distribution of workplace ostracism among nurses.

Although previous reviews have synthesized evidence on workplace ostracism, the existing literature has mainly focused on broader occupational populations and organizational behavior perspectives. Howard et al. (23) clarified the general antecedents and consequences of workplace ostracism in a cross-industry and cross-occupational organizational behavior review. Li et al. (24) mainly reviewed the effects of workplace ostracism on employee attitudes, wellbeing, and behaviors, while Li and Li (25) examined the influence of workplace ostracism on work behaviors within a specific cultural context. Although these studies provide an important foundation for understanding workplace ostracism, they did not focus on the healthcare field. Given the unique characteristics of healthcare settings, the manifestations, mechanisms, and practical consequences of workplace ostracism among nurses may differ from those observed in general occupational groups. To date, no comprehensive review has examined the current state of research on workplace ostracism among nurses. A scoping review is an effective approach for summarizing research progress, synthesizing current findings, and identifying knowledge gaps in a specific field. Therefore, this review adopted a scoping review methodology to map the literature on workplace ostracism in nursing, with a focus on the level, associated factors, and potential mechanisms of workplace ostracism, thereby providing evidence for future research and nursing management practice.

## 2 Methods

This study was conducted using the scoping review guidance of the Joanna Briggs Institute (JBI) as the methodological framework (26). The review was reported according to the Preferred Reporting Items for Systematic Reviews and Meta-Analyses extension for Scoping Reviews (PRISMA-ScR) (27). In alignment with open science principles, the project was pre-registered on the Open Science Framework with the following identifier:10.17605/OSF.IO/5QD3R.

### 2.1 Identifying the research questions

The research questions were developed based on a preliminary review of the literature. The following questions guided this scoping review:

- What has been reported about workplace ostracism in the field of nursing?
- How do existing studies describe the occurrence of workplace ostracism among nurses and its associated factors?
- What effects is workplace ostracism associated with in relation to nurses' individual, professional, and organizational outcomes?

### 2.2 Eligibility criteria

This review used the Population, Concept, and Context (PCC) framework to guide the search strategy and define the inclusion and exclusion criteria (28). Table 1 presents the eligibility criteria mapped to the PCC framework.

**Table 1:** Eligibility criteria based on the PCC framework.

PCC element	Inclusion criteria	Exclusion criteria
Population	Registered nurses or assistant nurses aged 18 years or older. Registered nurses were defined as nursing professionals who had passed a nationally or regionally recognized nursing qualification examination and obtained a nursing license. Assistant nurses were defined as individuals who had recently graduated from nursing school but had not yet obtained a nursing license.	Studies that did not involve nurses.
Concept	Studies that examined the current status of workplace ostracism among nurses or its related factors.	Studies that did not examine workplace ostracism as a core concept.
Context	Participants were working in healthcare-related settings, such as hospitals, medical institutions, or rehabilitation centers.	Studies conducted in non-healthcare workplace settings.

To ensure the reliability and rigor of the included evidence, this review only included studies published in peer-reviewed journals. Because the research team had limited resources for translating non-English studies, only studies published in English were included.

The exclusion criteria were as follows: review articles, such as systematic reviews, scoping reviews, narrative reviews, and meta-analyses; conference abstracts; duplicate publications; and studies for which the full text was not available.

### 2.3 Search strategy

An initial search was conducted in PubMed and Web of Science to identify studies on workplace ostracism in nursing. The titles, abstracts, and keywords of the retrieved studies were reviewed. Based on this analysis, the search strategy was developed.

The final search terms were grouped into three categories: nurses, workplace, and ostracism. Subject headings, free-text terms, and Boolean operators were combined in the search strategy. The following databases were searched: PubMed, Embase, Web of Science, APA PsycInfo, The Cochrane Library, Scopus, CINAHL, and MEDLINE Ultimate. Reference lists of the included studies were also screened using the snowballing method. The search covered the period from database inception to April 19, 2026. The detailed search strategy is provided in the Supplementary Table 1.

### 2.4 Study selection and data extraction

All retrieved records were imported into EndNote, and duplicates were removed. Two reviewers, Shiya Hu and Binfen Lin, independently screened the titles, abstracts, and full texts according to the inclusion and exclusion criteria. Any disagreement between the two reviewers was resolved through discussion or consultation with a third reviewer, Fenglan Lin. This review followed the JBI approach for data extraction and analysis. Two reviewers independently extracted the data. A third reviewer then cross-checked the extracted data to minimize bias.

The extracted data included author, year, country, study design, participants, sample size, measurement instruments, level of workplace ostracism, theoretical framework, the role of workplace ostracism as a variable in the study, associated factors, outcome variables, and main findings. Given that the purpose of a scoping review is to describe the scope of evidence and study characteristics, this review did not conduct a formal methodological quality appraisal of the included studies. However, to enhance the rigor of result interpretation, we recorded the main methodological characteristics of the included studies during data extraction, including study design, sampling method, data collection method, measurement instruments, and reported reliability and validity information. Further details are provided in Supplementary Table 1.

### 2.5 Data synthesis

Given the heterogeneity of the included studies in study design, measurement tools, variable roles, and reported outcomes, a narrative synthesis was conducted. The extracted data were organized using a framework-based approach according to study characteristics, measurement methods, theoretical frameworks, variable roles, associated factors, potential mechanisms, and outcomes. The role of workplace ostracism was categorized as an independent variable, mediating variable, or outcome variable. When workplace ostracism was examined as an independent variable, the outcomes were further grouped into individual, professional, and organizational domains. Two researchers independently completed the classification and synthesis, and disagreements were resolved through discussion or consultation with a third researcher.

## 3 Result

**Figure 1:**
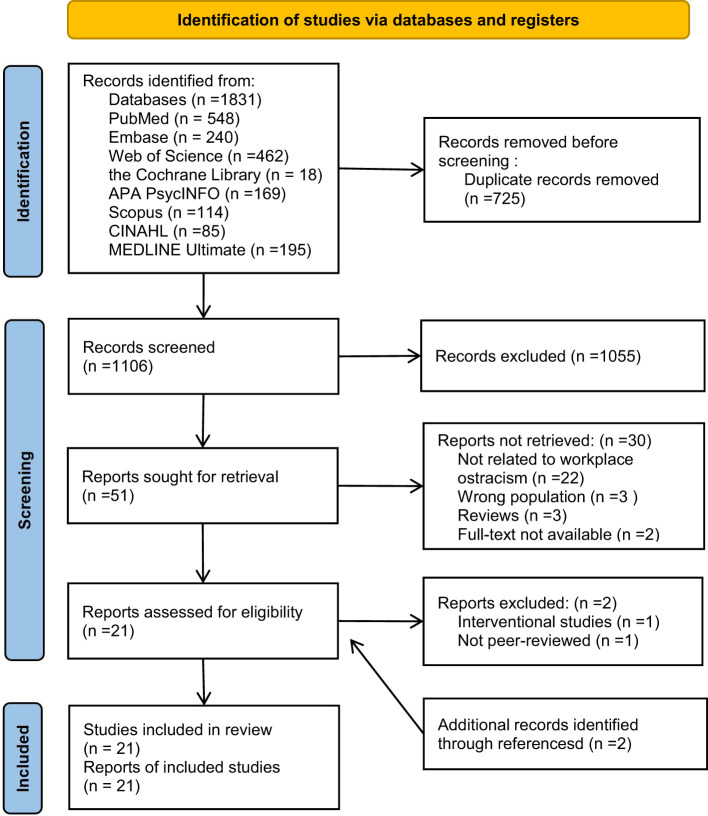
PRISMA flow diagram of the study selection process.

A total of 1,831 records were identified. After removing 725 duplicates, 1,106 records remained for title and abstract screening. These records were screened according to the inclusion and exclusion criteria. Of these, 51 records met the criteria for full-text review. In addition, two records were identified through reference list screening and were also assessed for full-text eligibility. Finally, 21 studies met the inclusion criteria and were included in this review. The study selection process is shown in Figure 1.

### 3.1 Basic characteristics

**Figure 2:**
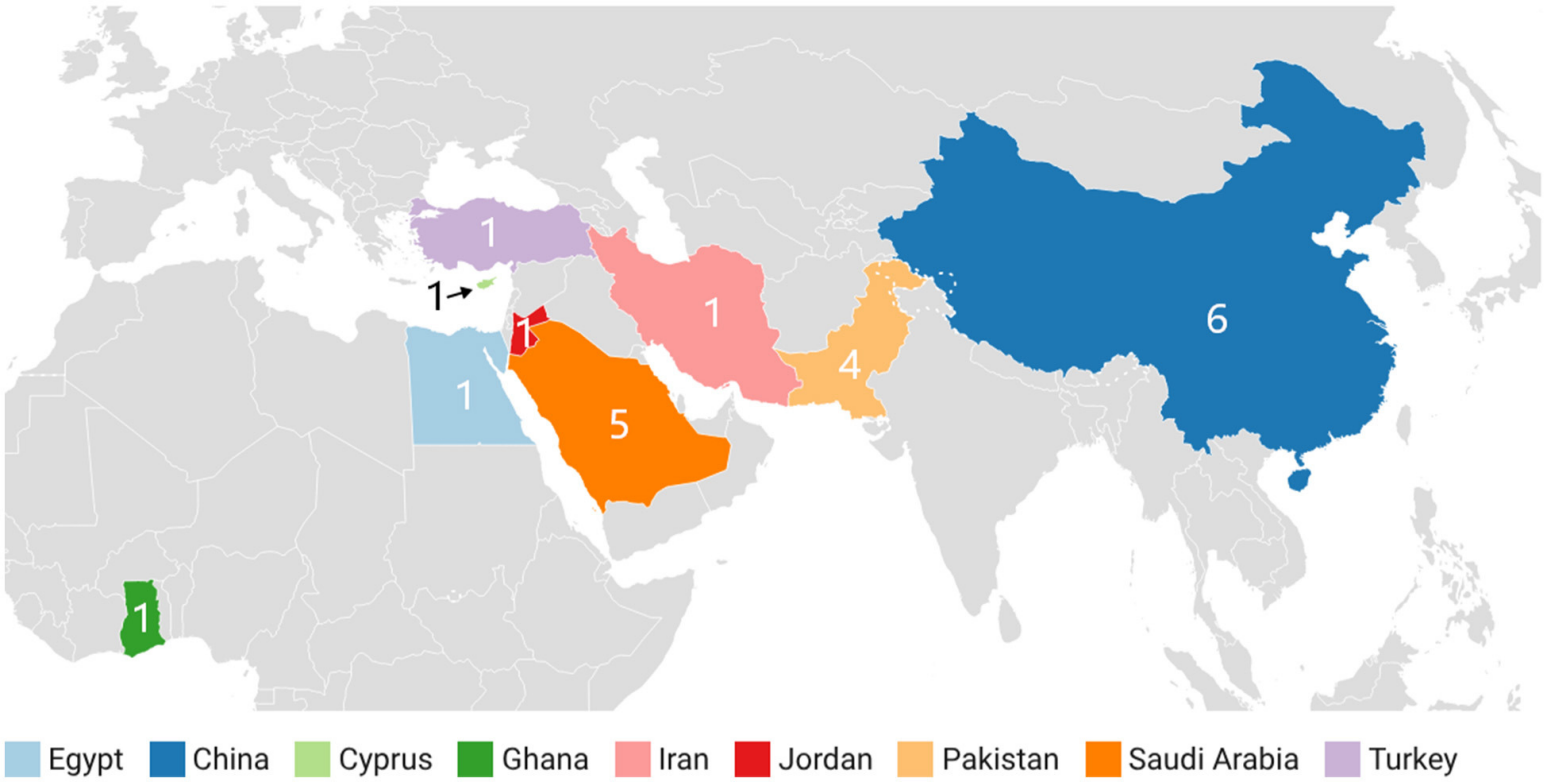
Geographical distribution of the included studies. The numbers indicate the number of publications from each country.

A total of 21 studies published between 2016 and 2026 were included. The included studies were conducted in nine countries. China contributed the largest number of studies, with six studies accounting for 28.6%, followed by Egypt with five studies accounting for 23.8%, and Pakistan with four studies accounting for 19.0%. The remaining studies were conducted in Cyprus, Saudi Arabia, Jordan, Ghana, Turkey, and Iran, with one study from each country, together accounting for 28.6%. The geographical distribution is shown in Figure 2. Among the 21 included studies, the participants were mainly clinical nurses (*n* = 18, 85.7%). Other participants included registered nurses (*n* = 1, 4.8%) (7), hemodialysis nurses (*n* = 1, 4.8%) (29), and intern nurses (*n* = 1, 4.8%) (10). Other basic characteristics are shown in Table 2.

**Table 2:** Characteristics of the included studies.

References	Origin	Study design	Participant	Measurement/Instrument	Workplace ostracism score	Outcome measurements	Variable name	Main conclusions
Gkorezis et al. (18)	Cyprus	Cross-sectional study	clinical nurses (*n* = 157)	(1) WOS^1^ (2) Organizational identification scale (3) Silence toward patient safety scale	2.01 ± 0.98^*a*^	workplace ostracism–organizational identification–silence toward patient safety	Workplace ostracism as an independent variable	Workplace ostracism positively predicted patient safety silence among nurses, and organizational identification played a partial mediating role in this relationship.
Sarfraz et al. (3)	Pakistan	Two-wave time-lagged quantitative survey study	clinical nurses (*n* = 241)	(1) WOS^1^ (2) Job stress (3) Perceived organizational support	3.43 ± 0.87^*a*^	workplace ostracism–stress	Workplace ostracism as an independent variable	Workplace ostracism was significantly positively associated with nurses' stress. Perceived organizational support moderated the relationship between workplace ostracism and stress, buffering the adverse effect of workplace ostracism on nurses' stress.
Qi et al. (9)	China	Three-wave time-lagged questionnaire survey	clinical nurses (*n* = 530)	(1) WOS^1^ (2) Hostile attribution bias scale (3) Emotional exhaustion scale (4) Unethical behavior scale	2.43 ± 1.51^*a*^	workplace ostracism–emotional exhaustion–unethical behavior	Workplace ostracism as an independent variable	Workplace ostracism positively predicted nurses' unethical behavior. Emotional exhaustion mediated this relationship, and hostile attribution bias moderated the effect of workplace ostracism on unethical behavior.
Sarwar et al. (11)	Pakistan	Cross-sectional study	clinical nurses (*n* = 217)	(1) WOS^1^ (2) Stress (3) Perceived organizational support scale (4) Customer service sabotage scale	3.41 ± 0.72^*a*^	workplace ostracism–stress–service sabotage behavior	Workplace ostracism as an independent variable	Workplace ostracism was associated with service sabotage behavior through increased stress. Perceived organizational support buffered the adverse effects of workplace ostracism and stress on service sabotage behavior.
Shafique et al. (16)	Pakistan	Cross-sectional study	clinical nurses (*n* = 417)	(1) WOS^1^ (2) Maslach burnout inventory (3) Organization-based self-esteem (4) Organizational identification (5) Workplace deviance (6) Ingratiation	4.07 ± 1.28^*a*^	workplace ostracism–deviant behavior	Workplace ostracism as an independent variable	Workplace ostracism was positively associated with nurses' deviant behavior. Organizational identification and organization-based self-esteem mediated this relationship, while ingratiation behavior buffered its adverse effects.
Gou et al. (12)	China	Two-wave time-lagged questionnaire survey	clinical nurses (*n* = 379)	(1) WOS^1^ (2) Emotional labor scale (3) Nurse-patient relationship scale (4) Turnover intention scale	/	workplace ostracism–emotional labor–nurse-patient relationship–turnover intention	Workplace ostracism as an independent variable	Workplace ostracism was positively associated with nurses' emotional labor, including both surface acting and deep acting. Surface acting reduced perceived nurse–patient relationship quality and further increased nurses' turnover intention.
Aliza et al. (36)	Pakistan	Three-wave time-lagged questionnaire survey	clinical nurses (*n* = 402)	(1) WOS^2^ (2) Emotional exhaustion scale (3) Employee negligence behavior scale (4) Task interdependence scale	1.83 ± 0.85^*a*^	workplace ostracism–emotional exhaustion–negligence behavior	Workplace ostracism as an independent variable	Workplace ostracism was positively associated with nurses' negligent behavior and emotional exhaustion. Emotional exhaustion mediated the relationship between workplace ostracism and negligent behavior.
Ali Awad And Mohamed El Sayed. (35)	Egypt	Cross-sectional study	clinical nurses (*n* = 340)	(1) WOS^2^ (2) Moral leadership questionnaire (3) Counterproductive work behaviors questionnaire	68.57 ± 10.59^*b*^; mean percent 60.71 ± 13.23^*b*^	workplace ostracism–counterproductive behaviors	Workplace ostracism as an independent variable	Post-COVID-19 workplace ostracism was positively associated with nurses' counterproductive work behavior. Ethical leadership was negatively associated with both workplace ostracism and counterproductive work behavior, and mitigated their adverse effects.
Qi et al. (30)	China	Three-wave time-lagged questionnaire survey	clinical nurses (*n* = 669)	(1) WOS^1^ (2) Cooperative and competitive orientation scale (3) Emotional dissonance scale (4) Regulation of emotions scale (5) Role job performance scale	2.26 ± 1.42^*a*^	workplace ostracism–emotional dissonance/regulation of emotions–in-role job performance	Workplace ostracism as an independent variable	Workplace ostracism was positively associated with emotional dysregulation and negatively associated with emotional regulation and in-role performance. Cooperative orientation strengthened emotional dysregulation, whereas competitive orientation promoted in-role performance through emotional regulation.
Ali et al. (34)	Saudi Arabia	Cross-sectional study	clinical nurses (*n* = 266)	(1) WOS^1^ (2) Innovative work behavior scale (3) Quality of nursing care scale	3.11 ± 0.42^*b*^	workplace ostracism–innovative work behavior–quality of nursing care	Workplace ostracism as an independent variable	Workplace ostracism among nurses was significantly negatively associated with innovative work behavior and nursing service quality. Innovative work behavior was positively associated with nursing service quality.
Attia et al. (32)	Egypt	Cross-sectional study	clinical nurses (*n* = 257)	(1) WOS^2^ (2) Emotional exhaustion scale (3) Defensive silence scale (4) Workplace deviance questionnaire	3.15 ± 0.54^*a*^	workplace ostracism–emotional exhaustion–defensive silence–deviant work behavior	Workplace ostracism as an independent variable	Workplace ostracism was positively associated with emotional exhaustion, defensive silence, and deviant workplace behavior. Emotional exhaustion and defensive silence mediated these relationships, with a chain mediating effect observed.
El-Sayed et al. (31)	Egypt	Cross-sectional study	clinical nurses (*n* = 352)	(1) WOS^1^ (2) Organizational silence scale (3) Active procrastination scale (4) Unintentional procrastination scale	3.44 ± 0.80^*a*^	workplace ostracism–organizational silence–active/passive procrastination behavior	Workplace ostracism as an independent variable	Workplace ostracism was positively associated with both active and passive procrastination. Organizational silence partially mediated the relationship between workplace ostracism and procrastination behavior.
Mrayyan And Algunmeeyn (7)	Jordan	Cross-sectional study	registered nurses (*n* = 408)	(1) WOS^1^ (2) Ethical leadership questionnaire (3) Counterproductive work behaviors questionnaire	3.68 ± 1.16^*a*^	workplace ostracism–counterproductive work behaviors	Workplace ostracism as an independent variable	Nurses reported a moderate level of workplace ostracism. Age and educational level predicted perceived workplace ostracism. Workplace ostracism was moderately positively associated with counterproductive work behavior, whereas ethical leadership was weakly negatively associated with counterproductive work behavior but not significantly associated with workplace ostracism.
Geng et al. (29)	China	Cross-sectional study	hemodialysis nurses (*n* = 434)	(1) WOS^1^ (2) Loneliness at work scale (3) Maslach burnout inventory (4) Career adapt-abilities scale	48.48 ± 1.28^*b*^	workplace ostracism–workplace loneliness–job burnout	Workplace ostracism as an independent variable	Workplace ostracism positively predicted job burnout. Workplace loneliness mediated this relationship, while career adaptability buffered the adverse effect of workplace loneliness on job burnout.
Liu et al. (33)	China	Cross-sectional study	clinical nurses (*n* = 346)	(1) WOS^2^ (2) Moral courage scale (3) Helping behavior scale (4) Employee resilience scale	2.76 ± 1.25^*a*^	observed workplace ostracism–moral courage–helping behavior	Workplace ostracism as an independent variable	Marital status influenced nurses' perceived workplace ostracism. Observed workplace ostracism was positively associated with helping behavior. Moral courage partially mediated this relationship, while employee resilience moderated the relationship between observed workplace ostracism and moral courage and its indirect effect.
Atinga et al. (10)	Ghana	Qualitative descriptive study	clinical intern nurses (*n* = 18)	Semi-structured interviews	/	linical placement ostracism–psychological distress; clinical placement ostracism–professional identity formation	Workplace ostracism as an independent variable	Nursing students experienced ostracism in clinical placements through emotional isolation, restricted professional access, devaluation, and lack of support. These experiences threatened belonging, self-esteem, control, and meaningful existence, and affected learning confidence, clinical engagement, and professional identity formation. Students mainly coped through avoidance, silence, or peer support.
Al-Atwi et al. (37)	China	Cross-sectional study	clinical nurses (*n* = 280)	(1) WOS^1^ (2) Paranoia scale (3) Team cognitive diversity scale (4) Service performance scale	1.91 ± 1.14^*a*^	paranoia–workplace ostracism–service performance	Workplace ostracism as a mediating variable	Paranoid tendency and team cognitive diversity positively predicted nurses' workplace ostracism. Workplace ostracism negatively predicted service performance and mediated the effects of paranoid tendency and team cognitive diversity on service performance.
Özkan et al. (19)	Turkey	Cross-sectional study	clinical nurses (*n* = 230)	(1) WOS^1^ (2) Abusive supervision scale (3) 4-item measure of experienced incivility (4) Workplace bullying scale	/	abusive supervision–workplace ostracism–turnover intention	Workplace ostracism as a mediating variable	Abusive supervision, workplace incivility, and workplace bullying were positively associated with nurses' turnover intention. Workplace ostracism partially mediated the relationship between abusive supervision and turnover intention.
Elliethey et al. (20)	Egypt	Cross-sectional study	clinical nurses (*n* = 369)	(1) WOS^2^ (2) Work ethics questionnaire (3) Counterproductive work behaviors questionnaire	20.87 ± 6.58^*b*^	work ethics–workplace ostracism–counterproductive work behaviors	Workplace ostracism as a mediating variable	Gender and educational level influenced nurses' perceived workplace ostracism. Work ethics were negatively associated with workplace ostracism and counterproductive work behavior, while workplace ostracism was positively associated with counterproductive work behavior. Workplace ostracism mediated the relationship between work ethics and counterproductive work behavior.
El-Guindy et al. (22)	Egypt	Cross-sectional study	clinical nurses (*n* = 100)	(1) WOS^2^ (2) Workplace incivility scale (3) Quality of nursing care scale	30.20 ± 4.73^*b*^	workplace incivility–workplace ostracism–quality of nursing care	Workplace ostracism as a outcomet variable	More than half of the nurses reported a low level of workplace ostracism. Workplace incivility was significantly and weakly positively associated with workplace ostracism among nurses.
Gharaei et al. (21)	Iran	Cross-sectional study	clinical nurses (*n* = 340)	(1) Workplace exclusion scale (2) Personal–social information questionnaire	36.63 ± 9.03^*b*^	employment status; university of education; incentive record; physical disorders; colleagues' envy; managers' discrimination	Workplace ostracism as a outcome variable	Nurses experienced a low level of workplace ostracism. Workplace ostracism was associated with employment status, educational level, history of receiving incentives, current physical illness, perceived coworker jealousy, and perceived managerial discrimination.

WOS^1^, original workplace ostracism scale; WOS^2^, self-modified workplace ostracism scale; ^*a*^Item-level Likert mean score; ^*b*^Total score; /, not reported or not applicable.

Most studies used a cross-sectional survey design (*n* = 20) (3, 7, 9, 11, 12, 16, 18–22, 29–37). Only one study used a qualitative design (10). Although cross-sectional studies predominated, there was some methodological heterogeneity. Specifically, one study (37) used multilevel nested sampling and multilevel analysis, one study used snowball sampling (19), and two studies (9, 32) used random sampling, whereas the remaining studies mainly used convenience sampling. In addition, five studies (3, 9, 12, 30, 36) adopted time-lagged designs to reduce common method bias and strengthen the interpretation of temporal ordering between variables. Further details are provided in Supplementary Table 1.

### 3.2 Measurement methods and reported levels of workplace ostracism in nursing

Regarding the measurement of workplace ostracism, 13 studies (3, 7, 9, 11, 12, 16, 18, 19, 29–31, 34, 37) used the 10-item Workplace Ostracism Scale developed by Ferris et al. (5). Six studies used modified versions of the original Workplace Ostracism Scale (20, 22, 32, 33, 35, 36). One study used the Workplace Exclusion Scale (21). The Cronbach's α coefficients reported in the included studies (3, 9, 12, 18, 20, 22, 29, 30, 32–37) Cronbach's α mostly ranged from 0.84 to 0.98, indicating good internal consistency of the relevant scales among nurses. Further details are provided in Supplementary Table 1. Because the included studies differed in measurement instruments, scoring methods, and reporting formats, this review did not conduct a pooled analysis or direct cross-study comparison of workplace ostracism levels. Existing studies suggest that nurses experience varying degrees of workplace ostracism, with most studies reporting low to moderate levels.

### 3.3 Theoretical frameworks of workplace ostracism in nursing

In terms of theoretical frameworks, the included studies showed a certain degree of theoretical diversity. Seven studies (7, 16, 18, 31, 32, 34, 35) used Social Exchange Theory to explain how workplace ostracism may be associated with nurses' outcomes, while six studies (3, 11, 12, 29, 31, 36) used Conservation of Resources Theory to explain the mechanisms through which workplace ostracism may be related to nurses' behaviors and professional outcomes. In addition, a small number of studies adopted other theoretical perspectives. Gkorezis et al. (18) also incorporated Belongingness Theory to examine the relationships among workplace ostracism, organizational identification, and nurses' silence toward patient safety. The specific theoretical frameworks used in the included studies are provided in Supplementary Table 1.

### 3.4 Factors associated with workplace ostracism

The factors associated with workplace ostracism among nurses can be described from individual and organizational perspectives (7, 20–22, 33, 37). First, demographic characteristics, including age (7), sex (20), educational level (7, 20), marital status (33), physical health status (21), and employment status (21), were identified as factors associated with workplace ostracism among nurses. In addition to demographic factors, personal traits (37) were also found to positively predict workplace ostracism among nurses, while work ethics may influence nurses' perceptions of workplace ostracism (20). Organizational factors are also important and cannot be overlooked in the nursing context. The included studies showed that workplace incivility (22), organizational silence (31) leaders' and colleagues' attitudes (21) were important organizational factors associated with workplace ostracism in nursing.

### 3.5 Workplace ostracism as an independent variable

Sixteen studies (3, 7, 9–12, 16, 18, 29–36) examined workplace ostracism as an independent variable and explored its associations with individual, professional, and organizational outcomes.

First, individual-level outcomes mainly involved nurses' psychological and emotional responses. Specifically, these included increased stress (3, 11), emotional exhaustion (9, 32, 36), job burnout (16, 29), workplace loneliness (29), psychological distress (10), and emotional dissonance (30).

Second, professional-level outcomes mainly involved nurses' work attitudes, work behaviors, and professional performance. Workplace ostracism was associated with a range of adverse professional outcomes, including silence toward patient safety (18), defensive silence (32), unethical behavior (9), service sabotage behavior (11), deviant work behavior (16, 32), negligent behavior (36), counterproductive work behavior (7, 35), procrastination behavior (31), and increased turnover intention (10, 12). In addition, workplace ostracism was also associated with reduced in-role job performance (29), innovative work behavior (34), quality of nursing care (34), and professional identity formation (10). Notably, one study found that observed workplace ostracism was positively associated with nurses' helping behavior, with moral courage playing a partial mediating role. This suggests that when nurses witness others experiencing workplace ostracism, they may engage in helping behavior driven by moral courage (33).

Organizational-level outcomes mainly involved the relationship between nurses and their organizations and the implications for organizational functioning. Relevant studies showed that workplace ostracism may reduce organizational identification and organization-based self-esteem (16, 18) and increase organizational silence (18, 31).

### 3.6 Workplace ostracism as a mediating variable

Three studies examined workplace ostracism as a mediating variable. Workplace ostracism mediated the relationships between paranoid tendency, team cognitive diversity, and service performance (37). It also partially mediated the relationship between abusive supervision and nurses' turnover intention (19). In addition, workplace ostracism mediated the relationship between work ethics and counterproductive work behavior (20).

### 3.7 Workplace ostracism as an outcome variable

One study examined workplace ostracism as an outcome variable (22). The results showed a significant weak positive correlation between workplace incivility and workplace ostracism among nurses.

## 4 Discussion

This scoping review provides a comprehensive synthesis of workplace ostracism among nurses. It focuses on the current evidence and potential mechanisms when workplace ostracism is examined as an independent variable, a mediating variable, and an outcome variable. The accumulated evidence suggests that workplace ostracism may be associated with nurses' individual health, professional performance, and organizational functioning through multiple pathways, including psychological resource depletion, reduced organizational identification, impaired communication, and negative work behaviors. Although cross-sectional studies predominated, some studies used time-lagged designs, multilevel nested analyses, and qualitative interviews, reflecting a certain degree of methodological diversity in research on workplace ostracism among nurses. However, this diversity also highlights the limitations of causal inference and longitudinal understanding.

### 4.1 Workplace ostracism as an independent variable

This review found that most studies examined workplace ostracism as an independent variable, suggesting that it may be associated with a wide range of individual, professional, and organizational outcomes among nurses. Compared with workplace violence, workplace ostracism is usually manifested as being ignored, excluded, isolated from information, or relationally distanced, and is therefore more subtle and ambiguous. Consequently, its adverse consequences may not appear immediately as overt conflicts, but may accumulate gradually through daily workplace interactions and affect nurses and nursing organizations through different pathways.

From the individual perspective, workplace ostracism was closely associated with negative psychological outcomes, such as stress, emotional exhaustion, workplace loneliness, and job burnout. This is consistent with the finding of Howard et al. (23), that workplace ostracism may impair individuals' psychological resources. Conservation of Resources Theory (13) suggests that continuous exposure to resource-threatening environments may activate resource-protection mechanisms and lead to sustained psychological resource depletion. Unlike some other occupations, nursing is highly dependent on teamwork and communication (38), making emotional resources particularly important for nurses. Therefore, when workplace ostracism weakens nurses' access to colleague support and emotional replenishment, it may be more likely to be associated with workplace loneliness, stress, and emotional exhaustion (39). In addition, this review found that emotional resource depletion is an important mechanism linking workplace ostracism with nurses' outcomes, with emotional exhaustion being one of the most frequently identified mediating factors. Three studies confirmed the mediating role of emotional exhaustion (9, 32, 36). This finding reveals a key pathway through which workplace ostracism may lead to negative work behaviors among nurses by depleting psychological resources. Emotional exhaustion reflects a state of psychological fatigue caused by long-term interpersonal stress and emotional resource depletion. It weakens nurses' ability to maintain emotional expression and work engagement in clinical practice (40).

From the professional perspective, workplace ostracism may not only affect nurses' psychological states but also further undermine their work attitudes, professional behaviors, and nursing practice performance. Studies have shown that workplace ostracism among nurses may increase employee silence (18, 32). One possible explanation is that when nurses feel marginalized or undervalued within the team, their psychological safety and willingness to speak up may decline. As a result, they may be more likely to remain silent to avoid further rejection or exclusion. For nursing organizations, silence is not merely an individual passive coping response; it may also hinder the timely flow of nursing information, delay the identification of care-related risks, and subsequently affect team decision-making and patient safety (41). Meanwhile, workplace ostracism may also trigger a range of negative professional behaviors by disrupting the reciprocal relationships between nurses and their organizations, teams, and colleagues. From the perspective of Social Exchange Theory (15), when nurses experience long-term neglect and exclusion from their work teams or organizations, their trust, sense of responsibility, and willingness to reciprocate may decline, which may be reflected in unethical behavior, service sabotage behavior, deviant work behavior, negligent behavior, counterproductive work behavior, and procrastination behavior. In addition, workplace ostracism may weaken nurses' positive professional performance, as reflected in its associations with reduced in-role job performance, innovative work behavior, quality of nursing care, nurse–patient relationship quality, and professional identity formation. These findings suggest that workplace ostracism may not only increase negative behaviors but also reduce nurses' motivation for active engagement, innovative practice, and maintaining high-quality nursing care.

From the organizational perspective, workplace ostracism may weaken nurses' connection with their organizations by undermining their need for belonging. Belongingness Theory (17, 42) suggests that individuals have a fundamental need to maintain stable social relationships and gain group acceptance. When nurses are ignored, excluded, or marginalized within their teams, their sense of organizational belonging and psychological safety may be threatened, which may further reduce organizational identification and organization-based self-esteem (16, 18) At the same time, reduced belongingness may decrease nurses' willingness to speak up, report risks, and participate in team activities, thereby increasing organizational silence (18, 31). For nursing organizations, these effects may further hinder information flow and team collaboration, with potential implications for quality of care and patient safety (41, 43). Therefore, workplace ostracism is not only an interpersonal stress experience at the individual level, but may also represent an important organizational risk that weakens nurses' organizational connectedness and the safety culture of nursing care.

It should be noted that the relationship between workplace ostracism and negative nurse-related outcomes may not be unidirectional. Because most included studies used cross-sectional designs, it remains unclear whether workplace ostracism is associated with stress, emotional exhaustion, burnout, silence behavior, and negative work behaviors, or whether nurses experiencing these difficulties are more likely to perceive or report workplace ostracism. Therefore, future longitudinal or cross-lagged studies are needed to clarify temporal ordering and causal pathways.

### 4.2 Workplace ostracism as a mediating and outcome variable

This review found that workplace ostracism mediated the relationships between paranoid tendency, team cognitive diversity, and service performance (37). It also partially mediated the relationship between abusive supervision and nurses' turnover intention (19). In addition, workplace ostracism mediated the relationship between work ethics and counterproductive work behavior (20). These findings suggest that workplace ostracism may act as an important psychological and relational mechanism. It may explain how individual characteristics, team structure, leadership style, and organizational ethics influence work outcomes among nurses.

In nursing contexts, the relationship between abusive supervision and workplace ostracism deserves particular attention. Abusive supervision may further increase nurses' turnover intention through workplace ostracism. This suggests that inappropriate managerial behavior may not only directly affect nurses' work attitudes. It may also indirectly harm nurses by shaping an exclusionary team climate (19, 44). Previous studies have also shown that destructive leadership styles can consume nurses' emotional resources and cause psychological distress. These factors are important contributors to nurse turnover (45). This mechanism helps explain the role of nursing management behavior in the formation of workplace ostracism. It also provides insight into the organizational sources of increased turnover intention among nurses.

This review also found a significant weak positive correlation between workplace incivility and workplace ostracism among nurses (22). Workplace incivility is a low-intensity but persistent form of negative interpersonal interaction. It may weaken team respect, coworker trust, and psychological safety. As a result, nurses may be more likely to feel ignored, excluded, or marginalized. Although the reported level of workplace ostracism in this study was low, workplace incivility was still significantly associated with workplace ostracism. This suggests that even subtle indifference, offense, or lack of respect may gradually damage nurses' sense of belonging and acceptance within the team.

### 4.3 Factors associated with workplace ostracism among nurses

Seven studies (20, 21, 30–33, 37) showed that workplace ostracism was associated with both individual and organizational factors. In terms of individual factors, age, gender, and educational level were important indicators. Male nurses and nurses with lower educational levels tended to report higher levels of workplace ostracism (20). However, the evidence on age was inconsistent. Mrayyan and Algunmeeyn (7) found that younger nurses reported higher perceived workplace ostracism. This may be because they were in the early stage of their careers. They may have limited clinical experience, organizational adaptability, and team relationship resources. Therefore, they may be more likely to feel ignored or marginalized in complex nursing work environments. In contrast, some studies did not find a significant association between age and workplace ostracism (20, 21). In addition, nurses with higher levels of paranoia may be more likely to interpret ambiguous interpersonal cues as intentional exclusion or negative evaluation, thereby intensifying their perceptions of being ignored or marginalized (37). Work ethics may also influence experiences of workplace ostracism. Nurses with stronger work ethics may be more likely to maintain fair and supportive team interactions, thereby reducing experiences of ostracism (20, 46).

Nursing organizational structures may contribute to the formation of workplace ostracism. First, nursing work is usually embedded in a clear hierarchical structure, in which nurse managers, senior nurses, and junior nurses may differ in power, experience, and access to resources (47). Task allocation, information transmission, and career development resources are often shaped by organizational hierarchy and team relationships. When abusive supervision, managerial discrimination, workplace incivility, colleague envy, or poor management of team cognitive diversity exists within nursing organizations, some nurses may be more likely to be excluded from communication and support networks, thereby developing stronger experiences of workplace ostracism. At the same time, organizational support and the organizational communication climate may determine whether the consequences of workplace ostracism are buffered or amplified. Perceived organizational support can buffer the adverse effects of workplace ostracism on nurses' stress and service sabotage behavior (3, 11), whereas organizational silence may restrict nurses' expression, help-seeking, and problem solving, thereby reinforcing negative behavioral responses after workplace ostracism (31). This suggests that workplace ostracism among nurses is not merely an individual interpersonal issue, but is closely related to power structures, resource allocation, communication climate, and support systems within nursing organizations. A positive, fair, and supportive organizational environment may help enhance nurses' psychological safety and sense of team belonging, thereby reducing experiences of workplace ostracism and its adverse consequences (48).

### 4.4 Cultural factors and nurses' perceptions of workplace ostracism

Cultural factors may influence how nurses perceive and respond to workplace ostracism. In Western contexts, workplace ostracism may be more readily understood as a violation of individual rights, a lack of organizational justice, or a threat to psychological safety (8). Western cultures generally place greater emphasis on individual autonomy, equal communication, and direct expression (49). Therefore, when nurses experience being ignored, excluded, or marginalized, they may be more likely to interpret such experiences as organizational injustice or negative workplace events and respond through formal communication, complaint mechanisms, or by seeking organizational support. However, in non-Western cultural contexts, such as China, Egypt, and Pakistan, perceptions of workplace ostracism may be more deeply embedded in social relationships, hierarchical order, and collective values. In the Chinese cultural context, gender-role perceptions within the nursing profession may affect the professional experiences of male nurses (50). Nursing has traditionally been viewed as women's work, and male nurses may face pressure related to professional identity and social recognition (51). When their professional role conflicts with social gender expectations, male nurses may be more likely to perceive marginalization, low recognition, and insufficient belonging within teams or organizational environments. Similarly, in some Middle Eastern healthcare settings, nurses' perceptions of workplace ostracism may not only involve gender, but may also be strongly shaped by hierarchical workplace structures and collectivist orientations, making workplace ostracism more social and relational in nature (20). Therefore, nursing managers should fully consider how cultural background may influence nurses' perceptions of ostracism, their ways of expressing such experiences, and their help-seeking behaviors when identifying and addressing workplace ostracism.

### 4.5 Implications for nursing management

This review highlights the complex role of workplace ostracism in nursing. In practice, organizational change and workplace competition are becoming more intense. Under these conditions, workplace ostracism may be difficult to eliminate completely. Therefore, nursing managers should pay greater attention to this issue. They should provide appropriate guidance and reduce its negative effects in nursing practice as much as possible.

First, a healthy work environment is essential for nursing practice. Nursing managers should recognize that ethical leadership is an important driver of nurses' positive attitudes and adaptive behaviors (52). Hospitals can provide ethical leadership training, communication skills training, and management accountability mechanisms. These strategies may help leaders serve as positive role models. They may also reduce the formation and spread of exclusionary interactions within nursing teams. Second, organizational support is an important protective factor. It may help reduce workplace ostracism and its adverse consequences (3, 21, 53). Nursing managers should improve the organizational environment at the system level by optimizing staffing, enhancing fairness in scheduling and task allocation, establishing safe and confidential reporting mechanisms, and regularly assessing team climate and psychological safety within nursing units. At the individual level, nurses should strengthen their communication skills. Effective communication is an important strategy for reducing negative workplace behaviors (54). In addition, positive psychological interventions are also important. One study showed that resilience training may help alleviate negative emotions caused by workplace ostracism (55).

### 4.6 Limitations

Several limitations should be acknowledged. First, only English-language studies were included because the research team lacked the capacity to translate non-English publications, which may have resulted in language bias. Second, most included studies were cross-sectional, and although some used time-lagged designs, the current evidence remains insufficient to establish causal relationships between workplace ostracism and nurses' psychological, behavioral, or organizational outcomes. Third, as this was a scoping review, no formal methodological quality appraisal was conducted; however, key methodological characteristics, including study design, sampling methods, and measurement instruments, were summarized to support interpretation of the findings. Fourth, many studies relied on nurses' self-reported questionnaires, which may be affected by social desirability, recall bias, and common method bias. Finally, the evidence base was geographically concentrated, with most studies conducted in China, Egypt, and Pakistan. Therefore, the findings should be generalized cautiously to other cultural and healthcare organizational contexts, and future research should include more multicenter, longitudinal, and cross-cultural studies.

## 5 Conclusion

This review synthesized the available evidence on workplace ostracism among nurses and provides a theoretical basis for nursing managers to improve the nursing work environment. The findings suggest that workplace ostracism, as a negative workplace experience, is associated with nurses' psychological and emotional states, professional performance, and organizational outcomes. Nursing managers should pay attention to the identification, prevention, and management of workplace ostracism. By creating a fair, supportive, and psychologically safe work environment, nursing organizations may reduce workplace ostracism and its potential adverse consequences, thereby promoting nurses' occupational health and the quality of nursing care.
