# Supplementary material for: Workplace ostracism in nursing: a scoping review
Source: Front Public Health. 2026 Jul 16;14:1884011. doi: 10.3389/fpubh.2026.1884011 (PMC13422499; doi:10.3389/fpubh.2026.1884011)
Supplement: Supplementary file 1 [file Table_1.docx]

**Supplementary Material A . Search Strategy.**

| **Electronic database** | **Query of keywords for search strategy** | **Results** |
| --- | --- | --- |
| PubMed | Search: #1 AND #2 Filters: Free full text, English, from 1998/1/1 - 2026/4/19 “nurses”［MeSH Terms］ OR “nurse practitioners” ［MeSH Terms］ OR “nurse*”［Title/Abstract］ OR “nurse practitioner*”［Title/Abstract］ OR “nursing personnel”［Title/Abstract］ OR “registered nurse*”［Title/Abstract］） AND (work*[Title/Abstract] OR "Workplace"[MeSH]) AND (ostracism[Title/Abstract] OR exclusion[Title/Abstract] OR discrimination[MeSH]) | 548 |
| Embase | ('nurse'/exp OR 'nurse':ti,ab OR 'nurse practitioner'/exp OR 'nurse practitioner*':ti,ab OR 'nursing personnel':ti,ab OR 'registered nurse*':ti,ab) AND ('workplace'/exp OR 'workplace':ti,ab) AND ('ostracism':ti,ab OR 'social exclusion'/exp OR 'exclusion':ti,ab OR 'discrimination'/exp) | 240 |
| The Cochrane Library | ((nurses):ti,ab,kw OR (nurse practitioners:ti,ab,kw OR (nurse*):ti,ab,kw OR (nurse practitioner*):ti,ab,kw OR (nursing personnel):ti,ab,kw) OR (registered nurse*):ti,ab,kw)) AND (workplace):ti,ab,kw) AND ((ostracism):ti,ab,kw OR (exclusion):ti,ab,kw OR (discrimination):ti,ab,kw) | 18 |
| Web of Science | **(TS=(nurses OR** nurse practitioners **OR** nurse* **OR** nurse practitioner* OR nursing personnel OR registered nurse***) ) AND (TS=workplace) AND TS=(**ostracism **OR** exclusion **OR** discrimination **)** Publication date from 1998/1/1 - 2026/4/19 | 462 |
| Scopus | (TITLE-ABS-KEY("nurses") OR TITLE-ABS-KEY("nurse practitioners") OR TITLE-ABS-KEY("nurse*") OR TITLE-ABS-KEY("nurse practitioner*") OR TITLE-ABS-KEY("nursing personnel")OR TITLE-ABS-KEY("registered nurse*")) AND (TITLE-ABS-KEY("**workplace**") AND TITLE-ABS-KEY("ostracism") OR TITLE-ABS-KEY("exclusion") OR TITLE-ABS-KEY("discrimination") ) | 114 |
| CINAHL | Search: S1 AND S2 AND S3 Filters: Free full text, English, from 1998/1/1 - 2025/4/19  **S1 nurses OR** nurse practitioners **OR** nurse* **OR** nurse practitioner* OR nursing personnel OR registered nurse*  **S2 workplace**  **S3** ostracism **OR** exclusion **OR** discrimination | 85 |
| APA PsycInfo | (nurses OR nurse practitioners OR nurse* OR nurse practitioner* OR nursing personnel OR registered nurse*) AND workplace AND (ostracism OR exclusion OR discrimination) | 169 |
| MEDLINE Ultimate | Search: S1 AND S2 AND S3 Filters: Free full text, English, from 1998/1/1 - 2025/4/19  **Title or abstract XB= nurses OR** nurse practitioners **OR** nurse* **OR** nurse practitioner* OR nursing personnel OR registered nurse*  **Title or abstract XB =workplace**  **Title or abstract XB=** ostracism **OR** exclusion **OR** discrimination | 195 |

**Supplementary Material B . Methodological characteristics of the included studies**

| **Author，year** | **Theoretical model** | **Study design** | **Study setting** | **Sampling methods** | **Data collection methods** | **Reliability/validity of measurement instruments** |
| --- | --- | --- | --- | --- | --- | --- |
| Gkorezis et al.(18)2016 | Social exchange theory, belonging theory | Cross-sectional study | Single-center | Convenience Sampling | Paper questionnaire | Cronbach’alpha correlation：0.93 |
| Sarfraz et  al.(3) 2019 | Conservation of Resources Theory | two-wave time-lagged quantitative survey study | Multicenter | Convenience Sampling | Paper questionnaire | Cronbach’s α： 0.89 - 0.97 |
| Qi et al.(9) 2020 | Affective Events Theory | Three-wave time-lagged questionnaire survey | Multicenter | Random sampling | Paper questionnaire | Cronbach’alpha correlation：0.98 |
| Sarwar et al.(11) 2020 | Conservation of Resources Theory; Equity Theory | Cross-sectional study | Single-center | Convenience Sampling | Paper questionnaire | Cronbach’alpha correlation：0.70 |
| Shafique et al.(16)2020 | Social Exchange Theory | Cross-sectional study | Multicenter | Convenience Sampling | Paper questionnaire | / |
| Gou et al.(12) 2022 | Conservation of Resources Theory; Emotion Regulation Theory | two-wave time-lagged quantitative survey study | Single-center | Convenience Sampling | Paper questionnaire | Cronbach’alpha correlation：0.84 |
| Aliza et al.(36) 2022 | Conservation of Resources Theory | Three-wave time-lagged questionnaire survey | Multicenter | Convenience Sampling | Paper questionnaire | Cronbach’alpha correlation：0.96 |
| Ali Awad And Mohamed El Sayed.(35) 2023 | Social Exchange Theory; | Cross-sectional study | Single-center | Convenience Sampling | Paper questionnaire | Cronbach’alpha correlation：0.94 |
| Qi et al.(30) 2025 | Cognitive Appraisal Theory of Stress | Three-wave time-lagged questionnaire survey | Multicenter | Convenience Sampling | Paper questionnaire | Cronbach’alpha correlation：0.98 |
| Ali et al.(34) 2025 | Social Exchange Theory; Equity Theory | Cross-sectional study | Multicenter | Convenience Sampling | Paper questionnaire | Cronbach’alpha correlation：0.86 |
| Attia et al.(32) 2025 | Social Exchange Theory | Cross-sectional study | Single-center | Random sampling | Paper questionnaire | Cronbach’alpha correlation：0.84 |
| El‐Sayed et al.(31) 2025 | Social Exchange Theory; Conservation of Resources Theory; Organizational Silence Theory | Cross-sectional study | Multicenter | Convenience Sampling | questionnaire collection after one-to-one interviews | / |
| Mrayyan And Algunmeeyn (7) 2025 | Social Exchange Theory; Social Learning Theory | Cross-sectional study | Multicenter | Convenience Sampling | online survey via Facebook and WhatsApp | / |
| Geng et al.(29) 2026 | Conservation of Resources Theory | Cross-sectional study | Multicenter | Convenience Sampling | Paper questionnaire | Cronbach’alpha correlation：0.93 |
| Liu et  al.(33) 2026 | Social Information Processing Theory | Cross-sectional study | Multicenter | Convenience Sampling | online questionnaire distributed via the Wenjuanxing platform | Cronbach’alpha correlation > 0.90 |
| Atinga et al.(10) 2026 | Temporal Need-Threat Model of Ostracism | Qualitative descriptive study | / | Purposeful sampling | / | / |
| Al-Atwi et al.(37) 2021 | Victim Precipitation Theory | Cross-sectional study | Single-center | multilevel nested sampling and multilevel analysis | Paper questionnaire | Cronbach’alpha correlation：0.98 |
| Özkan et al.(19)2022 | Social Learning Theory | Cross-sectional study | Single-center | Snowball sampling | data collected via email | / |
| Elliethey et al.(20) 2024 | / | Cross-sectional study | Single-center | Convenience Sampling | Paper questionnaire | Cronbach’alpha correlation：0.94 |
| A. El-Guindy et al.(22) 2022 | / | Cross-sectional study | Single-center | Convenience Sampling | Paper questionnaire | Cronbach’alpha correlation：0.89 |
| Gharaei et al.(21)2022 | / | Cross-sectional study | Multicenter | Convenience Sampling | Paper questionnaire | Cronbach’s α values for the coworker, supervisor, and language subscales were 0.88, 0.74, and 0.64, respectively |
